# Combined microbiome and metabolome analysis of gut microbiota and metabolite interactions in chronic spontaneous urticaria

**DOI:** 10.3389/fcimb.2022.1094737

**Published:** 2023-01-11

**Authors:** Zhen Luo, Zhangsi Jin, Xiaoran Tao, Ting Wang, Panling Wei, Caihong Zhu, Zaixing Wang

**Affiliations:** ^1^ Department of Dermatology, The First Affiliated Hospital of Anhui Medical University, Anhui, China; ^2^ Institute of Dermatology, Anhui Medical University, Anhui, China

**Keywords:** chronic spontaneous urticaria, allergy, pathogenesis, gut microbiota, serum metabolites, correlation analysis

## Abstract

**Background:**

The pathogenesis of chronic spontaneous urticaria (CSU) is unclear, and it turned out to be involved in biological processes, such as autoimmunity, autoallergy, inflammation, and coagulation. The gut microbiota plays an important role in immune and inflammatory diseases. However, the relationship between chronic spontaneous urticaria and the gut microbiota remains unknown.

**Methods:**

The stool and serum samples were taken from 15 CSU patients and 15 normal controls. Changes in the composition of gut microbiota and serum metabolism in CSU patients and normal controls were analyzed by 16S ribosomal RNA (rRNA) gene sequencing and untargeted metabolomics.

**Results:**

The results of 16S rRNA gene sequencing showed that compared with normal controls, CSU patients had increased α-diversity of gut microbiota and significant differences in β-diversity. At the phylum level, the relative abundance of *Firmicutes* increased and the relative abundance of *Bacteroidetes* and *Proteobacteria* decreased in CSU patients compared with healthy controls. At the genus level, six kinds of bacteria were significantly enriched in CSU patients and five in normal controls. Metabolomic analysis revealed altered levels of metabolites such as unsaturated fatty acids and purines. Correlation analysis of gut microbiota and metabolites showed that *Lachnospira* was negatively correlated with arachidonic acid, and *Gemmiger* was also negatively correlated with (±)8-HETE.

**Conclusion:**

This study suggests that changes in gut microbiota and metabolites may play a role in immune and inflammatory pathways in the pathogenesis of CSU patients.

## 1 Introduction

Chronic Spontaneous urticaria (CSU) is defined as wheals, angioedema, or both for more than 6 weeks without a clear predisposing factor (Zuberbier et al., 2022). The incidence of CSU is 0.1%-1.5%, which seriously affects both adults and children, with the former being more prevalent in women (Fricke et al., 2020). Approximately 80% of patients recover within 1 year, while more than 10% have illnesses that last for up to five years or more (Stepaniuk et al., 2020), and may relapse within months or years after remission. The cost on patients and society is considerably increased by the recurring course of CSU.

The pathogenesis of urticaria is primarily due to the activation of mast cells and basophils to degranulate and release histamine and other proinflammatory mediators that induce stimulation of sensory nerves, vasodilation and plasma extravasation, and inflammatory cell recruitment. However, the reasons for the activation of mast cells and basophils in CSU patients are complicated. There is increasing evidence that different physiological and pathological reactions including autoimmunity (Saini, 2014; Kolkhir et al., 2022), autoallergy (Hatada et al., 2013; Cugno et al., 2018), inflammation (Tedeschi et al., 2010; Kasperska-Zajac et al., 2015; Kolkhir et al., 2018) and coagulation (Tedeschi et al., 2014) are involved in the above-mentioned cell activation process leading to the formation of wheals.

The gut microbiota is considered to be a new metabolic organ that plays a crucial role in immunity and metabolism, and its metabolites have induction and intervention effects on host immunity and inflammation. In the past, researchers recognized the significance of gut microbiota in immune-mediated diseases such as inflammatory bowel disease (Glassner et al., 2020), type 2 diabetes (Karlsson et al., 2013), systemic lupus erythematosus (Katz-Agranov and Zandman-Goddard, 2017), atopic dermatitis (Paller et al., 2019). In 2017, Nabizadeh et al, for the first time, found that *Akkermansia muciniphila*, *Clostridium leptum* and *Faecalibacterium prausnitzii* levels were different relative to healthy controls, suggesting changes in the gut microbiota in chronic urticaria (Nabizadeh et al., 2017). In 2020, Wang et al. found that changes in gut microbiota were related to unsaturated fatty acid and butyrate metabolic pathways in CSU, providing a new research direction for the pathogenesis of CSU (Wang et al., 2020). However, the specific mechanism between CSU and gut microbiota remains unclear.

Here, we performed 16S gene sequencing and untargeted metabolomics on CSU and normal controls, and obtained the differences in gut microbiota and metabolism through joint analysis, which provided more data for the study of CSU gut microbiota.

## 2 Materials and methods

### 2.1 Patient information and sample collection

This study recruited 15 patients and 15 normal controls (NCs), aged 18-60 years, who were diagnosed with CSU in the First Affiliated Hospital of Anhui Medical University between March 2020 and July 2020. The diagnosis of CSU was established according to the EAACI/GA²LEN/EuroGuiDerm/APAAACI guideline (Zuberbier et al., 2022). Basic information such as age, gender, body mass index (BMI), place of residence and clinical symptoms were recorded. Exclusion criteria: use of antibiotics, probiotics, herbal medicines, steroidal drugs, immunosuppressive agents and biologics within 1 month; hemorrhoidal attacks, diarrhea and constipation within 1 month; allergies, diabetes, gastrointestinal disorders, autoimmune diseases, hypertension or systemic diseases known to seriously affect vital organs; pregnancy, lactation; induced urticaria (except dermographism). The patients were all on long-term oral antihistamines and had a recurring course of more than three months without clinical remission. All samples and clinical data were obtained with the informed consent of the research subjects. At the same time, this study was approved by the Ethics Review Committee of the First Affiliated Hospital of Anhui Medical University and in accordance with the Declaration of Helsinki.

Feces and serum from the same subjects were collected on the same day while fasting. The study subjects self-collected stool samples after defecation in the hospital and immediately transferred them to laboratory for cryopreservation. Blood samples were centrifuged at 3000 rpm for 10 minutes at room temperature, and the supernatant was collected and transferred to cryotubes. Serum samples with any hemolysis were excluded from the study. All fecal and serum samples were quickly frozen in liquid ammonia for 30s after collection, and then transferred to -80°C for storage.

### 2.2 Fecal DNA extraction and 16S sequencing

The genomic DNA of microbial samples in feces was extracted by CTAB/SDS method. The forward primer (5′-ACT CCT ACG GGA GGCAGC AG-3′) and reverse primer (5′-GGA CTA CHV GGG TWT CTA AT-3′) were used to amplify the 16S rRNA gene V3–V4 variable region from the bacterial DNA by PCR. The PCR products were purified with VAHTS™ DNA Clean magnetic beads (Vazyme Biotech, China). Finally, the library was sequenced on an Illumina MiSeq platform (Illumina, California, USA), and 250 bp/300 bp paired-end reads were generated. The raw sequencing data is saved in FASTQ format, and each deduplicated sequence generated after quality filtering, denoising, merging and removing chimeras is called ASVs (amplicon sequence variants) by the DADA2 method. Using the QIIME2 (2019.4) software, select the Greengenes database (Release 13.8) to perform species annotation on the characteristic sequences of each ASVs, and analyze the Alpha diversity and Beta diversity of the samples. Principal coordinates analysis (PCoA) was performed with the R programming language (Version 3.5.1). Linear discriminant analysis effect size (LEfSe) method was used to identify the biomarkers with statistical difference between CSU patients and NCs (Segata et al., 2011).

### 2.3 Serum sample collection, metabolite extraction, and UHPLC-MS/MS analysis

After the serum samples were thawed, they were vortexed for 10s to mix well, 50μL of the samples were transferred into a centrifuge tube, 300μL of 20% acetonitrile methanol internal standard extraction solution was added, and vortexed for 3min, centrifuged at 12,000r/min for 10min at 4°C. After centrifugation, 200μL of the supernatant was removed and placed in a -20°C refrigerator for 30min, and then centrifuged again at 12000r/min for 3min at 4°C. Pipette 180μL of the supernatant and place it in a liquid chromatography-tandem mass spectrometry (LC-MS/MS) system for metabolomic analysis.

Liquid chromatography–tandem mass spectrometry(LC-MS/MS) was performed using an ultra-high-performance liquid chromatography (UHPLC) system (1290 Infinity II LC System, Agilent Technologies, CA, USA) and an ultraperformance liquid chromatography (UPLC) high-strength silica (HSS) T3 column (1.8 mm, 2.1 mm × 100 mm) coupled to a quadrupole time-of-flight (Q-TOF) mass spectrometer (6545 LC/Q-TOF MS, Agilent Technologies). The samples were analyzed in both positive and negative ion modes. Mobile phase A used in positive ion mode was 0.1% formic acid in water, and in negative mode it was 5 mmol/L ammonium acetate in water. Mobile phase B was acetonitrile. The elution gradient was set as follows: 5% B at 0 min, 90% B at 11 min, 90% B at 12 min, and 5% B at 12.1 min, 5% B at 14 min. The flow rate was set to 0.4 mL/min. The source conditions of the electrospray ionization were set as follows: spray voltage was 2.5kV in positive mode and -1.5kV in negative mode; sheath gas flow rate was 11L/min; auxiliary gas flow rate was 8L/min; atomized gas voltage was 40V; sheath temperature was 325°C.

The acquired MS raw data were converted into mzML format using ProteoWizard software and processed by XCMS. Data preprocessing steps include peak identification, peak alignment, peak extraction, retention time correction, and peak integration. Peak areas were corrected using the “ support vector regression (SVR)” method, and peaks with a missing rate > 50% in each group of samples were filtered. The remaining peaks were determined by comparing their retention time and mass-to-charge ratio (m/z) with databases including HMDB database (http://www.hmdb.ca), KEGG database (http://www.genome.jp/kegg) and an internal online database. Univariate analysis was performed using Student’s t-test to detect the changes of metabolites in CSU patients. At the same time, we conducted multivariate statistical analysis, including principal component analysis (PCA) and orthogonal partial least squares-discriminant analysis (OPLS-DA), to understand the metabolite differences between CSU patients and NCs.

### 2.4 Statistical analysis

Microbial diversity was analyzed using QIIME2 software (Caporaso et al., 2010). α-diversity includes: Chao1 and Observed species indices to characterize richness, Shannon and Simpson indices to characterize diversity and Pielou’s evenness index to characterize evenness. The significance of diversity was calculated using the Kruskal-Wallis test. β-diversity was determined by the Jaccard, Bray-Curtis distance matrix calculated by QIIME2, and its significance was determined by PerMANOVA (permutational multivariate analysis of variance). The larger the Jaccard and Bray-Curtis distances, the smaller the similarity between microbial communities. LEfSe analysis found a statistically significant difference in gut microbiota between the two groups, with LDA values>3 and p-values <0.05 considered significant differences. For metabolomics, the Variable Importance in Projection (VIP) value>1 in the OPLS-DA analysis, and P<0.05 in the univariate analysis were considered to be significantly changed metabolites. Spearman correlation coefficient analysis was performed between the differential gut microbiota obtained by 16S rRNA analysis and the differential metabolites obtained by metabolomic analysis.

## 3 Results

### 3.1 Characterization of participants

A total of 15 CSU patients and 15 normal controls were recruited in this study. No significant differences were found in age, sex ratio, and BMI between the two groups (p>0.05) (**Supplementary Table S1**). The subjects who participated at the same time all lived in the same city for a long time, and there was no obvious mobility. Some clinical features of CSU patients are provided in **Supplementary Table S1**.

### 3.2 Altered gut microbiota diversity in CSU patients

**Figure 1:**
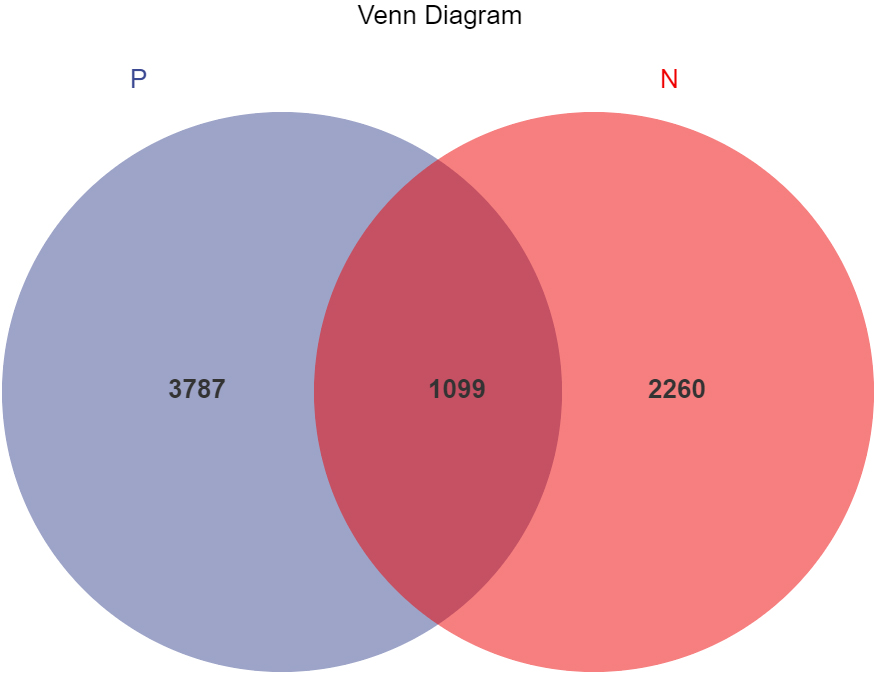
Venn diagram of ASVs (amplicon sequence variants) distribution in CSU group and normal controls group. A total of 7146 ASVs were obtained, including 3787 in the CSU group, 2260 in the normal controls group, and 1099 in the overlapping part of the two groups. P, CSU patients (blue); N, normal controls (red).

**Figure 2:**
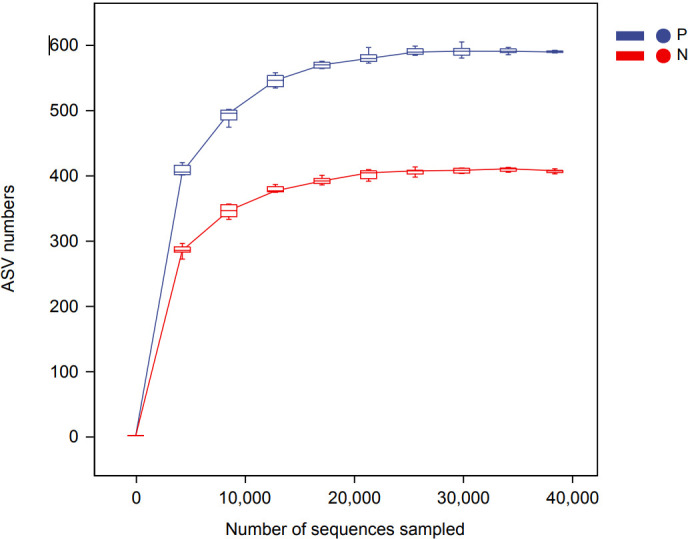
Rarefaction curve based on ASVs (amplicon sequence variants) count in normal controls and CSU patients. The number of ASVs increased with the deepening of sequencing depth and finally plateaued, indicating that the current sequencing depth was sufficient for community identification. P, CSU patients (blue); N, normal controls (red).

In this study, a total of 2,214,279 valid 16S sequencing markers (average 79,081) were obtained from 30 fecal samples, with a minimum of 65,594 per sample. A total of 7146 ASVs (**Supplementary Table S2**) were obtained after DADA2 processing, taxonomic annotation, and extraction with a minimum sample sequence size of 95% (Kemp and Aller, 2004), including 3787 in the CSU group, 2260 in the NCs group, and 1099 in the overlapping part of the two groups (**Figure 1**). The rarefaction curve showed that the number of ASVs increased with the deepening of sequencing depth and eventually leveled off, indicating that the current sequencing depth was sufficient for community identification **(** **Figure 2**).

**Figure 3:**
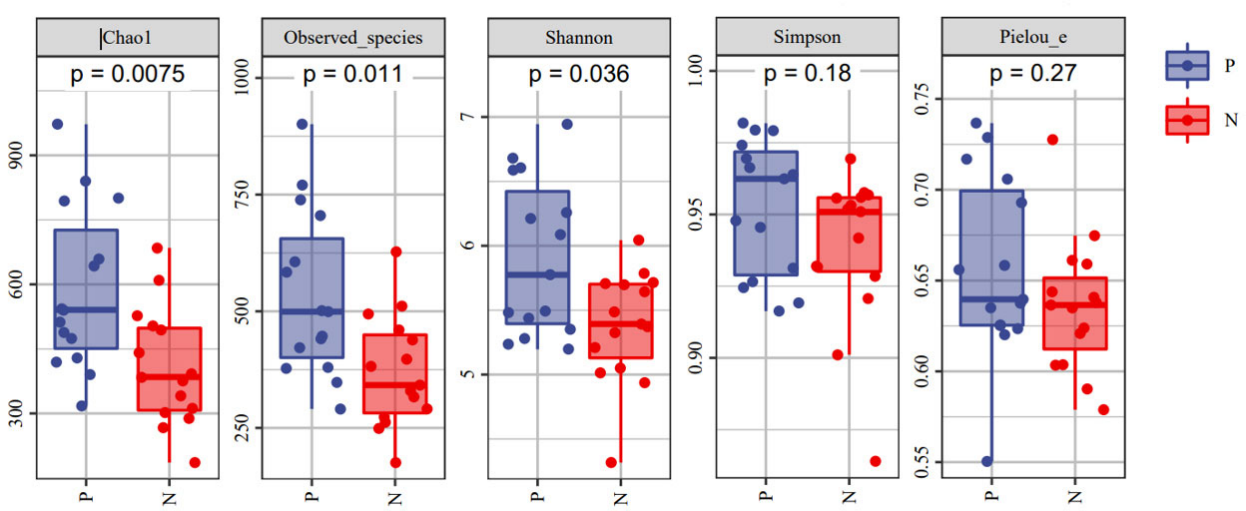
The α-diversity of CSU patients and normal controls was measured with the Chao1 and Observed species index, Shannon and Simpson index, Pielou’s evenness index. Chao1 and Observed species indices to characterize richness, Shannon and Simpson indices to characterize diversity and Pielou’s evenness index to characterize evenness. P, CSU patients (blue); N, normal controls (red).

**Figure 4:**
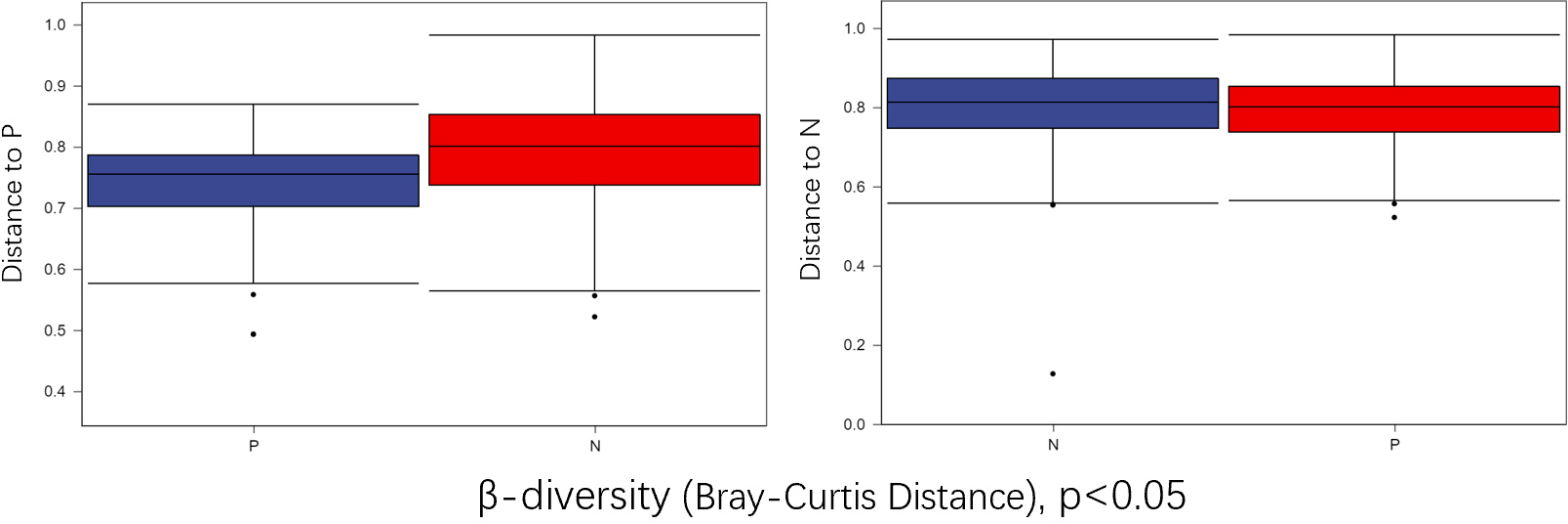
Group differences in β-diversity (Bray-Curtis distance index) with respect to CSU patients and normal controls. Statistical analyses were performed using PerMANOVA (permutational multivariate analysis of variance) (p < 0.05). P, CSU patients (blue); N, normal controls (red).

To comprehensively assess the changes in gut microbial diversity between CSU patients and NCs, we used six indices to analyze the alpha-diversity of the samples, namely Chao1 and Observed species index, Shannon and Simpson index, Pielou’s evenness index (**Figure 3**). Chao1 and Observed species were statistically different, Shannon was statistically different, but Simpson and Pielou’s evenness were not. It can be seen that the microbial α-diversity of the CSU group is higher than NCs. For β-diversity, Jaccard and Bray-Curtis distance matrix PCoA analysis was performed, and according to PerMANOVA analysis we obtained a significant difference in Jaccard and Bray-Curtis distances (**Figure 4**) between CSU and NCs group (**Supplementary Table S3**), confirming that CSU patients changes in microbial community structure.

**Figure 5:**
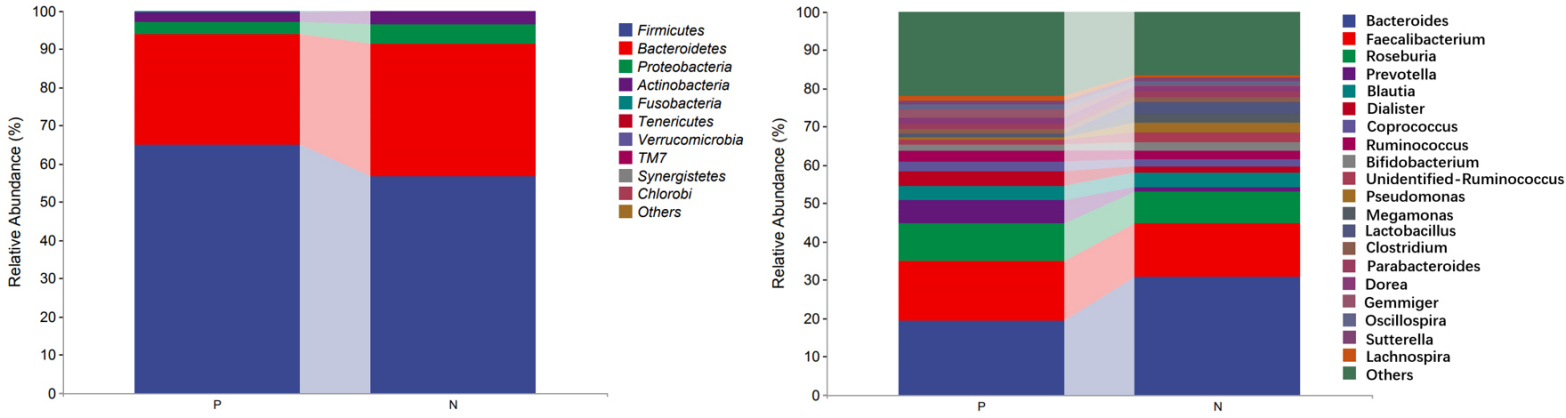
Profiles of the relative abundance at the phylum level (left) and the genus level (right) between the two groups. Only the top 10 phyla and the top 20 genera are shown. P, CSU patients; N, normal controls.

In the comparison of the abundance of microbiota in different taxonomy, it was found that the two groups of gut microbiota were mainly composed of *Firmicutes*, *Bacteroidetes*, *Proteobacteria* and *Actinobacteria*. *Firmicutes* was the predominant gut microbiota, accounting for 64.98% and 56.70% of CSU patients and NCs, respectively. At the phylum level, the relative abundance of *Firmicutes* increased and the relative abundances of *Bacteroidetes* and *Proteobacteria* decreased in CSU patients compared with NCs (**Figure 5**, **Supplementary Table S4**). At the genus level, we observed that the relative abundance of *Faecalibacterium*, *Roseburia*, *Prevotella*, *Dialister*, *Coprococcus*, *Gemmiger*, *Oscillospira* and *Lachnospira* was increased in patients with CSU, whereas the relative abundance of *Bacteroides*, *unidentified-Ruminococcus*, *Pseudomonas*, *Megamonas* and *Lactobacillus* was decreased in this group compared with NCs **(** **Figure 5**, **Supplementary Table S5**).

**Figure 6:**
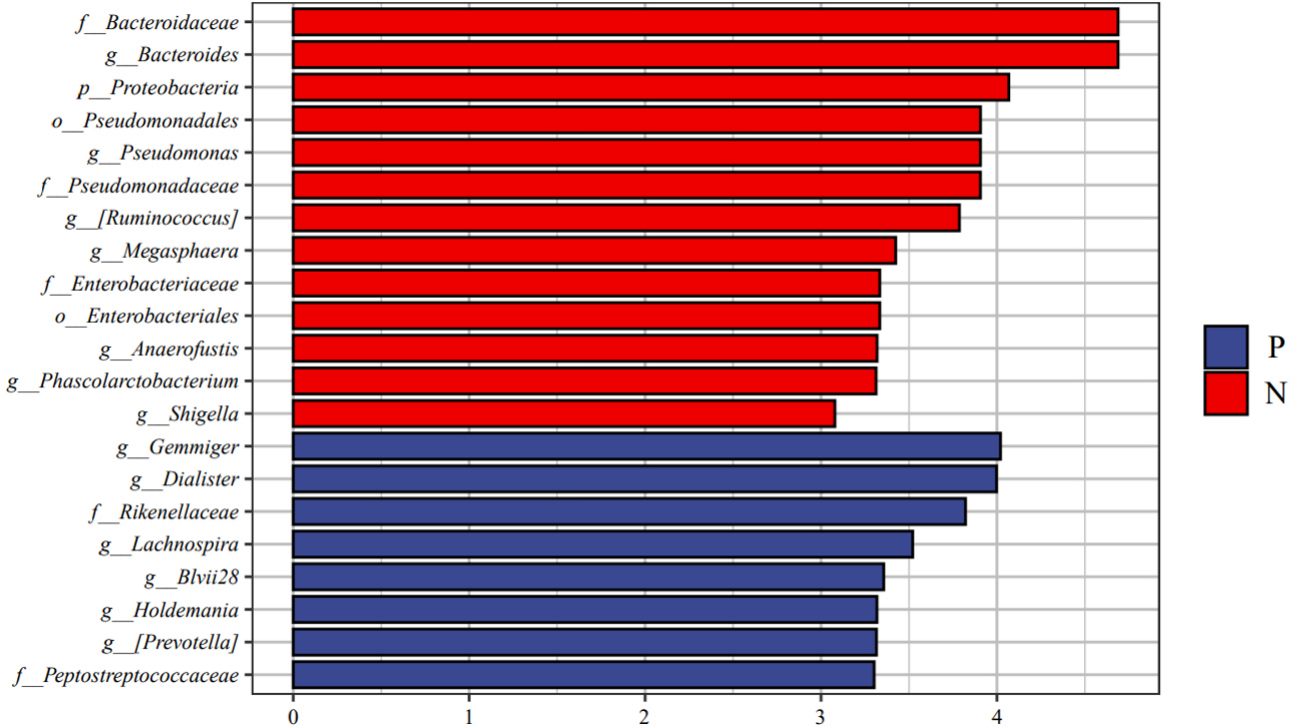
Linear discriminant analysis effect size (LEfSe) analysis was performed on the bacterial taxa relative abundance values between the two groups. Bacterial with linear discriminant analysis (LDA) score >3.0 and P < 0.05 were considered to be significantly differences. P, CSU patients (blue); N, normal controls (red).

**Figure 7:**
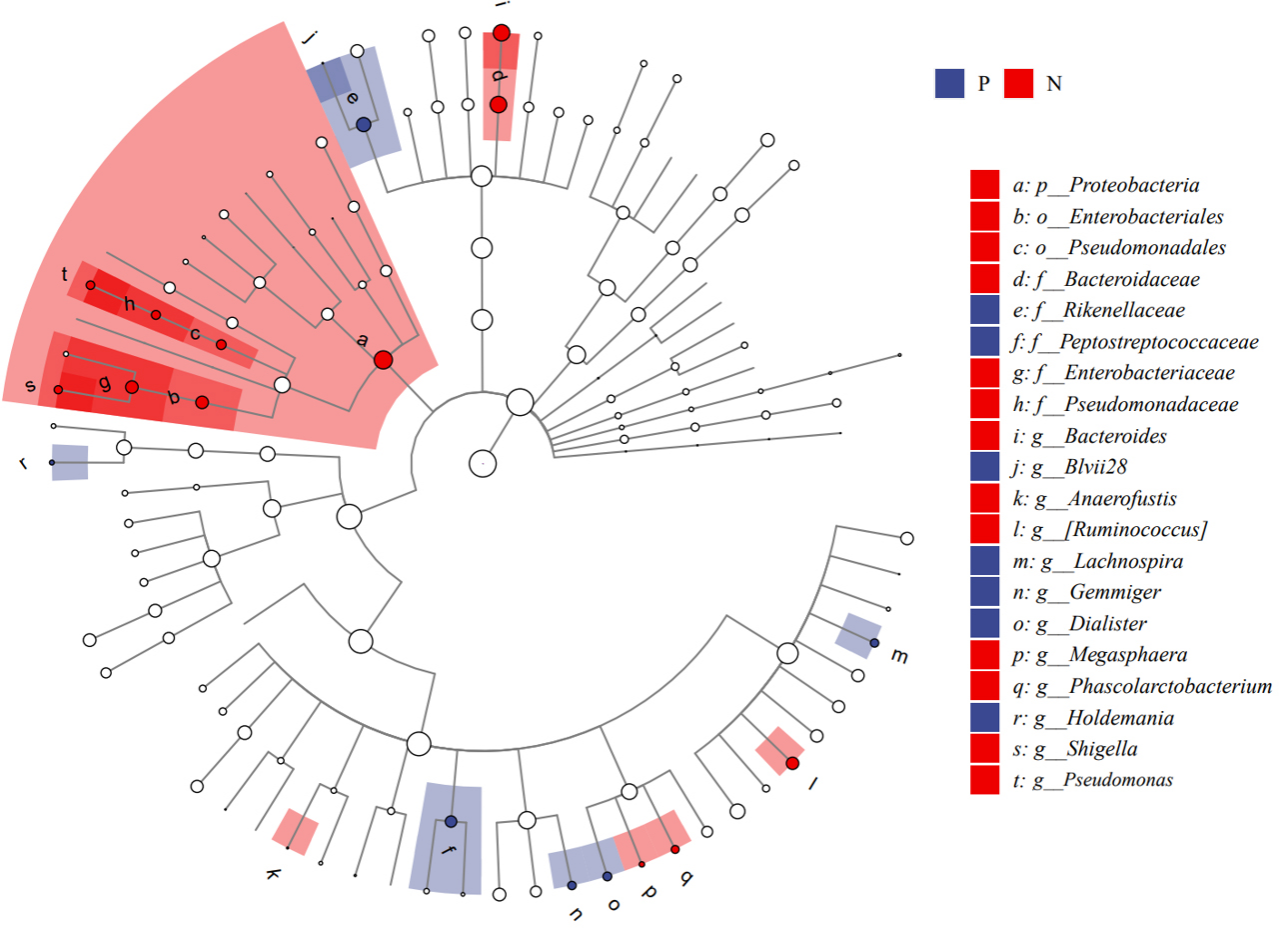
Cladogram of Linear discriminant analysis effect size (LEfSe) analysis of microbiome from 16S rDNA sequencing of normal controls and CSU patients. P, CSU patients (blue); N, normal controls (red).

We performed LEfSe analysis to further discover differences in gut microbiota between CSU and NCs (**Figure 7**, **Supplementary Table S6**). In comparison to normal controls. At the phylum level, the abundance of *Proteobacteria* in the feces of CSU patients was decreased. At the family level, *Rikenellaceae* and *Peptostreptococcus* were increased in CSU patients, while *Bacteroidaceae*, *Enterobacteriaceae*, and *Pseudomonadaceae* were decreased. At the genus level, *Gemmiger*, *Dialister*, *Lachnospira*, *Holdemania*, *unidentified-Prevotella*, *Blvii28* were increased in CSU patients, while *Bacteroidaceae*, *Pseudomonas*, *unidentified-Ruminococcus*, *Megasphaera*, *Anaerofustis*, *Shigella* were decreased (**Figure 6**). According to the LEfSe analysis of the Cladogram (**Figure 7**), the differences between the two groups of *Proteobacteria* are mainly concentrated in *Enterobacteriales* (*Enterobacteriaceae*, *Shigella*) and *Pseudomonadales* (*Pseudomonadaceae*, *Pseudomonas*) are opportunistic pathogens. Meanwhile, we used the R language and PICRUSt2 platform to predict the metabolic function of the differential microbiota through the KEGG database, and no significant metabolic pathways were detected (**Supplementary Table S7**).

### 3.3 Changes of intestinal metabolites

**Figure 8:**
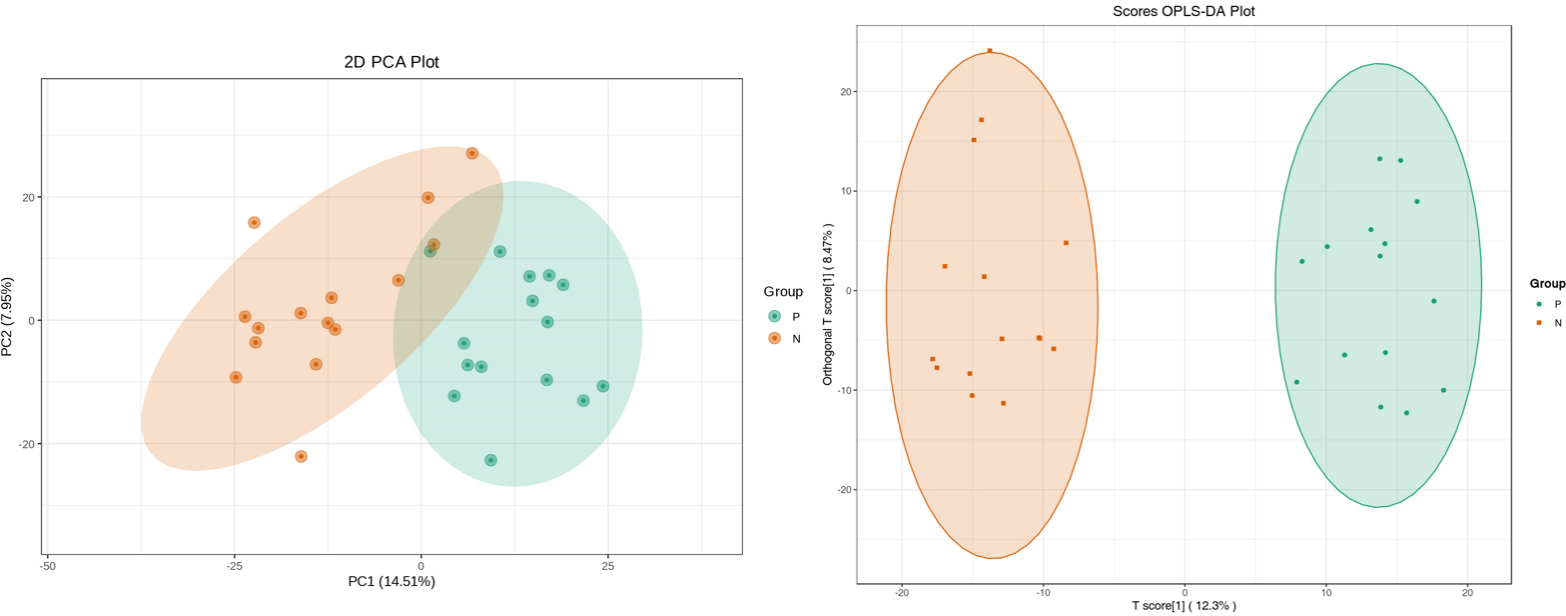
Principal component analysis (PCA) of fecal metabolomic data from CSU patients and normal controls. PCA shows clear separation of metabolic profiles between two groups. Orthogonal partial least squares discrimination analysis (OPLS-DA) of fecal metabolomic data from CSU patients and normal controls, the CSU group and the NCs group exhibited different clustering with R^2^Y of 0.956 and Q^2^ of 0.767. P, CSU patients (green); N, normal controls (yellow).

**Figure 9:**
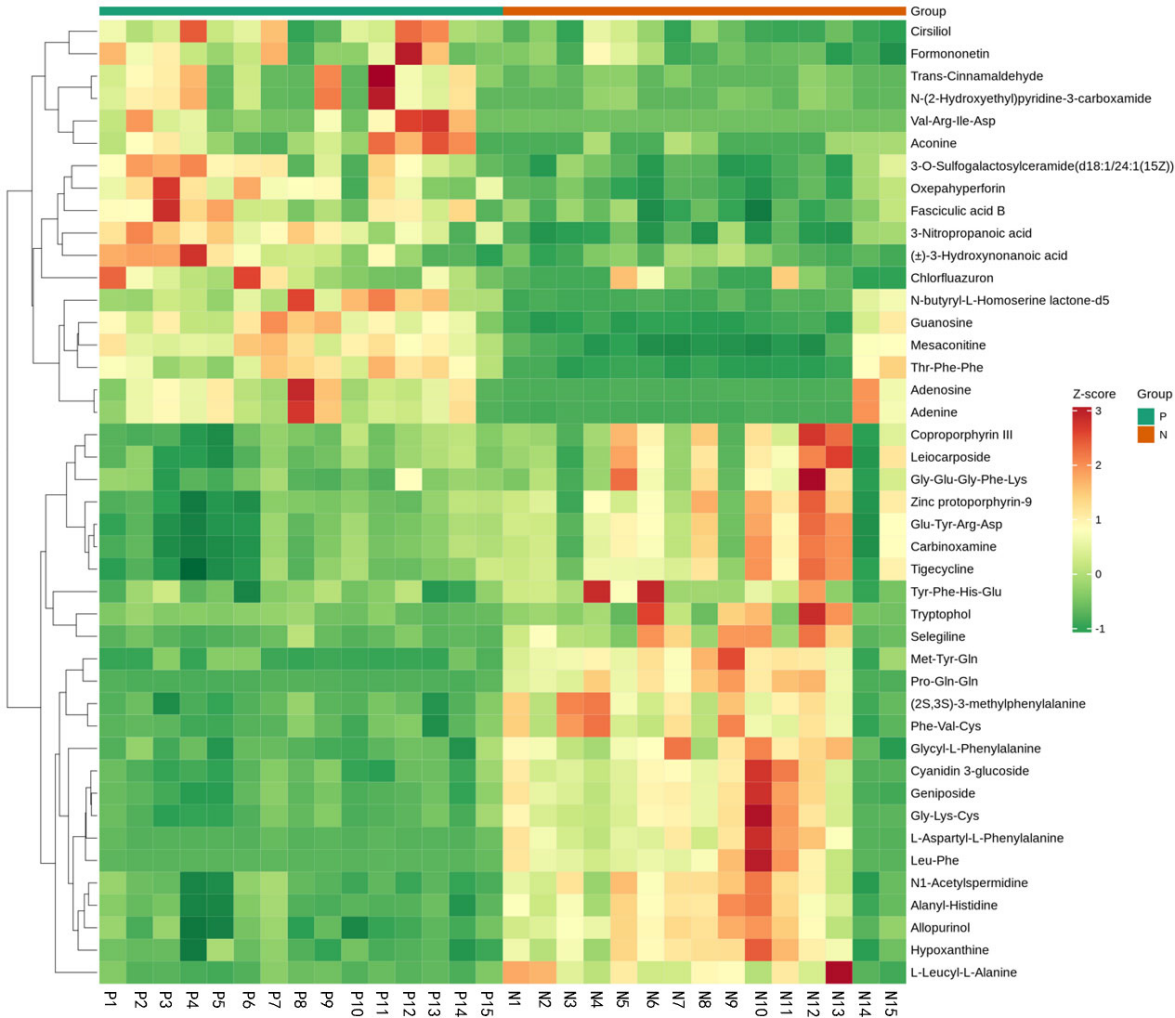
Fecal metabolic patterns in CSU patients and normal controls shown as a heatmap. Rows represent data for metabolites and columns represent the subjects. Red and green colors represent increased and decreased levels, respectively, of metabolites in patients with CSU compared to those in normal controls.

**Figure 10:**
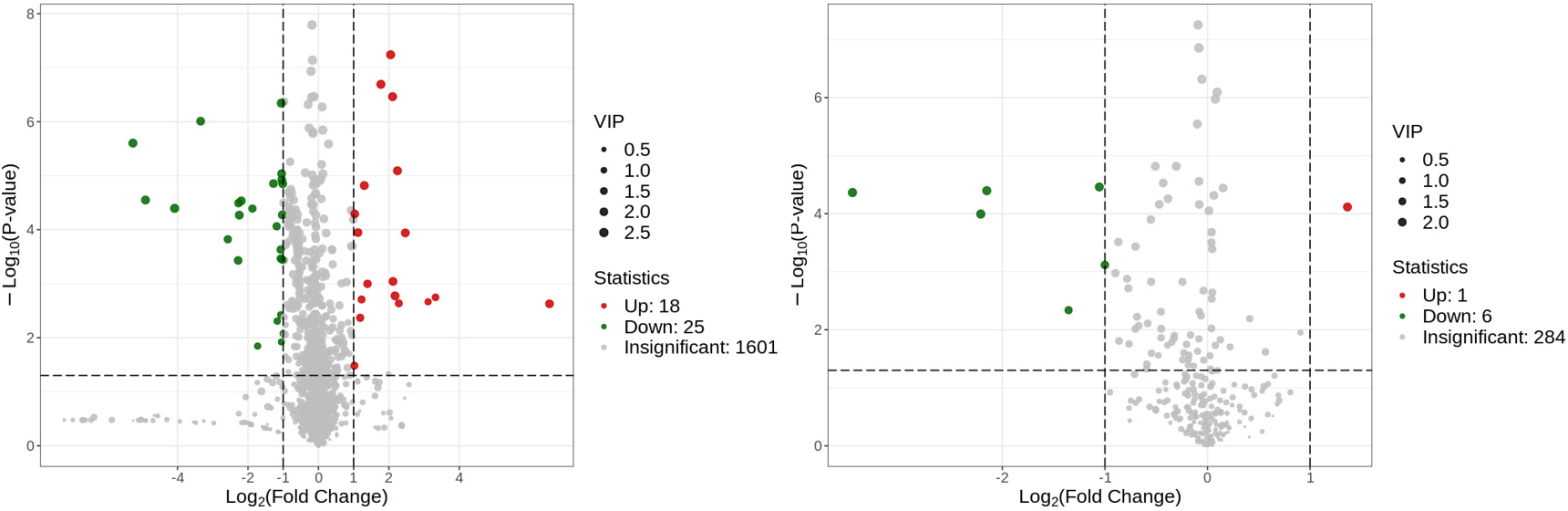
Volcano plot of the identified metabolites in positive ion mode (left) and negative ion mode (right). There were 43 differential metabolites in positive ion mode and 7 differential metabolites in negative ion mode.

Metabolites and fermentation products of gut microbiota can enter the bloodstream and affect human physiology. Therefore, we analyzed the serum metabolites of the CSU group and NCs by LC-MS/MS metabolomics to further discover the interaction between the various microbiota and the host. PCA shows clear separation of metabolic profiles between CSU patients and NCs. OPLS-DA showed that the CSU group and the NCs group exhibited different clustering (**Figure 8**), with R^2^Y of 0.956 and Q^2^ of 0.767, indicating that the model was valid, and the two groups exhibited different metabolic activity, demonstrating the presence of multiple metabolic pathways. According to the screening criteria of OPLS-DA model VIP>1 and t-test p<0.05, the differential metabolites with biological significance were mined. The bigger the value, the greater the contribution of the variable to the grouping. We hierarchically clustered the expression of differential metabolites, and drew a heatmap according to the relative metabolite content (**Figure 9**). We screened out a total of 50 differential metabolites. In the positive ion mode, there were 43 differential metabolites in the two groups, of which 18 were up-regulated and 25 were down-regulated; in the negative ion mode, there were 7 differential metabolites in the two groups, of which 1 was up-regulated and 6 were down-regulated (**Figure 10**). These metabolites include unsaturated fatty acids ((±)8-HETE, gamma-Linolenic acid, arachidonic acid), amino acids (leucine, phenylalanine), purine and other nucleotide metabolites (adenine, adenosine, inosine), etc. Unsaturated fatty acids were all down-regulated, while purine and other nucleotide metabolites were up-regulated (**Supplementary Table S8**).

**Figure 11:**
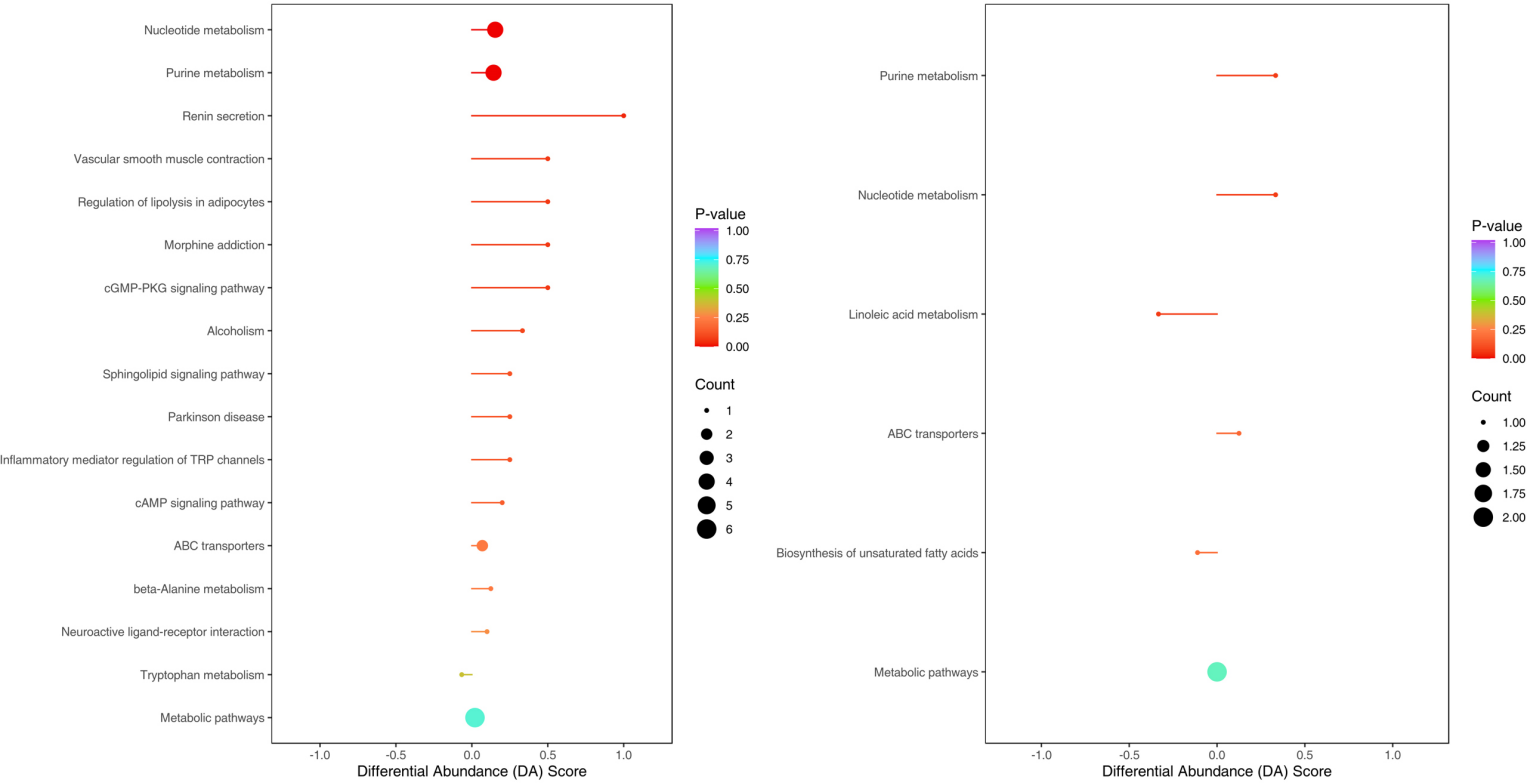
Metabolites were annotated into different metabolic pathways in positive ion mode and negative ion mode by KEGG enriched bubble chart based on KEGG. The abundance analysis showed that the KEGG pathway associated with the two fatty acids presented a negative differential score.

The enriched metabolic pathways were screened by KEGG pathway analysis. The top 20 metabolic pathways in positive and negative ion mode are shown in **Figures 11**. These pathways mainly include purine metabolism, nucleotide metabolism, unsaturated fatty acid biosynthesis, linoleic acid metabolism, renin secretion, vascular smooth muscle contraction, cGMP- PKG signaling pathway, etc. The abundance analysis showed that the KEGG pathway associated with the two fatty acids presented a negative differential score.

### 3.4 Conjoint analysis

**Figure 12:**
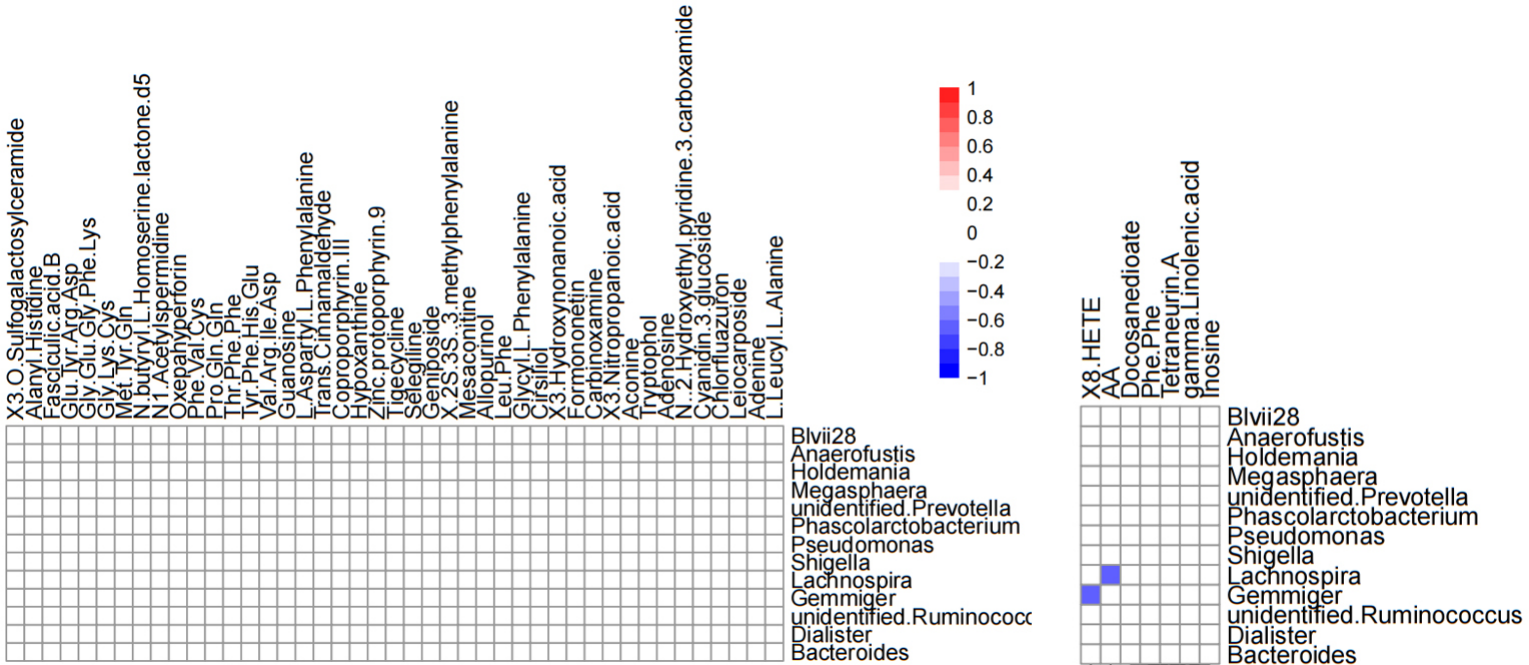
Heatmap of differential gut microbiota correlations between serum metabolites and genus levels in positive ion mode (left) and negative ion mode (right). Only significant correlations (p ≤ 0.05) are colored. Positive correlations are indicated in red and negative correlations in blue.

Correlation analysis was performed to better understand the relationship between the microbiome and the plasma metabolome. The altered metabolites screened by serum metabolomics analysis and the genus-level differential gut microbiota screened by 16S rRNA gene sequencing analysis were subjected to Spearman correlation analysis, and the criterion for statistical significance was the correlation coefficient |r|>0.60, p<0.05. We only found that *Lachnospira* was negatively correlated with arachidonic acid, and *Gemmiger* was negatively correlated with (±) 8-HETE. (**Figure 12**).

## 4 Discussion

Our study confirmed changes in gut microbiota and serum metabolic profiles in CSU patients. We attempted to search for possible pathogenesis and potential biomarkers of CSU by combining 16s gene sequencing and untargeted metabolomics.

This study explored the microbial diversity of the gut microbiota in CSU and NCs. α-diversity was used to compare the diversity within a sample, and we found that the two groups of microorganisms were statistically different in richness (Chao1 and Observed species) and diversity (Shannon), but not different in evenness (Pielou’s evenness). The α-diversity of the CSU was generally higher than that of the control group, which contradicted earlier research (Lu et al., 2019; Wang et al., 2020; Wang et al., 2021; Liu et al., 2021; Yüksekal et al., 2022). Some studies reported that there were significant differences in α-diversity, and the species diversity of chronic urticaria group decreased (Lu et al., 2019; Wang et al., 2020); other studies found no significant difference in α-diversity between CSU patients and controls (Zhang et al., 2021; Wang et al., 2021). This result may be controversial, and we think that it may be related to factors such as sample size, the geographical location of the subjects, eating habits, and research methods. While β-diversity was used to assess diversity across various samples, there was a significant difference between the CSU and NCs group, which is consistent with previous studies (Lu et al., 2019; Wang et al., 2020; Wang et al., 2021; Liu et al., 2021; Zhang et al., 2021; Yüksekal et al., 2022). Composition analysis of the two groups of samples showed that the relative abundance of *Firmicutes* increased in the CSU group, while the relative abundance of *Bacteroidetes* and *Proteobacteria* decreased, which was similar to the study by Wang et al (Wang et al., 2021), but different from the decreased abundance of *Firmicutes* in the study by Wang et al (Wang et al., 2020). Through LEfSe analysis, we further found that the variations in gut microbiota between two groups were mainly concentrated at the genus level, so we speculated that the changes in the microbiota associated with CSU may be mainly at the lower taxonomical level.

Our study found that *Bacteroides*, *unidentified-Ruminococcus, Megasphaera*, and *Anaerofustis* in the CSU group decreased compared with the control group. The decline of *Bacteroides* is consistent with some studies (Lu et al., 2019; Wang et al., 2020). *Bacteroides*, as the main component of the gut microbiota, is the main producer of short-chain fatty acids (SCFAs), mainly acetic and propionic acids. It has been demonstrated that *Bacteroides* has a regulatory effect on human immunity, mainly through its production of capsular polysaccharide A and SCFAs. Capsular polysaccharide A can maintain and regulate immune system homeostasis and prevents bacterial and viral infections (Zafar and Saier, 2021). Both acetate and propionate are effective anti-inflammatory mediators, which can inhibit the release of pro-inflammatory cytokines from neutrophils and macrophages, while SCFAs play an important role in various immune regulatory pathways such as inducing regulatory T cell differentiation, enhancing IL-10 production and inhibiting Th17 cells (Smith et al., 2013). Decreased abundance of *unidentified-Ruminococcus*, consistent with previous study (Wang et al., 2020). Some studies also found that the abundance of *Ruminococcaceae* decreased in infants with eczema, and inflammatory cytokines such as IL-6 and TNF-α increased, and a decrease in the number of *Ruminococcus* can induce a toll-like receptor inflammatory response (Arshi et al., 2014; West et al., 2015). These may confirm that inflammation is associated with CSU. In addition, *Ruminococcus* can also metabolize to produce SCFAs. *Megasphaera* are normal bacteria in the mouth, intestines and vagina that can metabolize to produce valerate. Valerates are also SCFAs, some studies have found that it may increase the cytotoxic activity of CD8^+^T cells and enhance the immune activity of the body (Luu et al., 2021). *Anaerofustis* is a gram-positive anaerobic bacteria that can produce acetate and butyrate from glucose (Finegold et al., 2004). Growing research proves that CSU is an autoimmune disease, and SCFAs play an important role in regulating and maintaining human immune function. Therefore, the reduction of some SCFAs-producing bacteria can lead to the occurrence of CSU.

We also found that *Gemmiger*, *Dialister*, *Lachnospira*, *Holdemania*, *unidentified-Prevotella* in the CSU group were higher than those in the control group. One study found that *Gemmiger* was consistently abundant in children with eczema across different modes of delivery (vaginal or caesarean section) or feeding type (infant formula or breastfeeding) (Zheng et al., 2016), consistent with our findings of increased abundance in the gut of CSU patients, suggesting that *Gemmiger* may have a facilitating role in allergic reactions. Our finding of increased abundance of *Dialister* in CSU patients contradicts a study of the gut microbiota of infants with food allergies, which found that children with allergic symptoms had lower numbers of *Dialister* than controls (Savage et al., 2018), and we speculate that this may be due to age differences in our study subjects. However, another study found that *Dialister*, as a microbial marker, was positively correlated with ankylosing spondylitis disease activity score in ileal and colon biopsies, and was more abundant in the acute inflammatory phase (Tito et al., 2017), suggesting that *Dialister* is closely related to the inflammatory reaction. As CSU is an inflammatory disease and *Dialister* abundance increases, there may be a certain correlation between the two. *Lachnospira* belongs to *Clostridiales* and has been found to be a microorganism that produces SCFAs. One study found that *Lachnospiraceae* and its subgroups were the major differences in gut microbiota between antihistamine responders and non-responders in CSU patients, with *Lachnospira* more abundant in antihistamine responders than non-responders (Liu et al., 2022). Our study did not distinguish between the efficacy of antihistamines in patients, so further studies are needed to address the increased abundance of *Lachnospira* in our study. Some studies have found that *Holdemania* is related to the occurrence of inflammatory response (Jang et al., 2020; Barandouzi et al., 2020), and CRP, IL-6, TNF-α and other inflammatory factors are elevated in CSU patients (Kasperska-Zajac et al., 2015; Kolkhir et al., 2018), so there may be a certain relationship between the two. Similarly, studies in mice have demonstrated that *Prevotella* can promote inflammatory diseases, and can promote Th17 immune response and neutrophil recruitment to induce chronic inflammation, so *Prevotella* may also have a role in promoting inflammation in CSU. In conclusion, changes in gut microbiota may promote or induce the occurrence of CSU in terms of inflammation and immunity.

Metabolites of gut microbiota are normally secreted in the gut and enter the circulatory system *via* the intestinal barrier, and are very important regulators of host metabolism (Canfora et al., 2019), so analyzing metabolite differences helps us discover the underlying pathological mechanisms of CSU. Through metabolomics analysis, we found that the metabolite differences between the CSU group and the control group were mainly concentrated in unsaturated fatty acids ((±)8-HETE, gamma-Linolenic acid, arachidonic acid), and they were all in a downward trend. At the same time, KEGG pathway analysis also showed that the unsaturated fatty acid metabolism pathway was down-regulated. This finding is consistent with the study by Wang et al, who also found that down-regulation of docosahexaenoic acid and arachidonic acid was positively associated with decreased abundance of *Bacteroides* (Wang et al., 2020). However, our study did not find a significant correlation between *Bacteroides* and unsaturated fatty acids, but suggested that *Lachnospira* was negatively correlated with arachidonic acid. Recent studies have shown that infants receiving formula plus long-chain unsaturated fatty acids such as arachidonic acid have a lower risk of allergic disease and respiratory disease than those receiving formula alone, and have better immune maturation (Birch et al., 2010; Lapillonne et al., 2014), suggesting that unsaturated fatty acids may have a protective effect on allergy. Unsaturated fatty acids such as arachidonic acid, eicosapentaenoic acid, and docosahexaenoic acid are also present in the cell membrane of immune system cells and affect immune function through various interaction mechanisms (Miles et al., 2021). Meanwhile, unsaturated fatty acids were found to have anti-inflammatory effects (Calder and Grimble, 2002). Therefore, the reduction of unsaturated fatty acids may aggravate or induce inflammation and allergic reactions. In addition, arachidonic acid can produce inflammatory mediators (prostaglandins, leukotrienes and related metabolites), which regulate the activity of inflammatory cells, the production of cytokines and the balance of Th1 and Th2, which is crucial to the occurrence and resolution of inflammation (Calder and Grimble, 2002; Das, 2021). Our study suggests that arachidonic acid and *Lachnospira*, (±) 8-HETE and *Gemmiger* are all negatively correlated, (±)8-HETE is one of the six monohydroxy fatty acids produced by non-enzymatic oxidation of arachidonic acid. 16s rRNA sequencing revealed that *Lachnospira* and *Gemmiger* abundances were elevated in the CSU group. Therefore, we speculate that the increase in the abundance of *Lachnospira* and *Gemmiger* may lead to the reduction of unsaturated fatty acids such as arachidonic acid, resulting in the imbalance of the immune and inflammatory systems, thereby promoting or inducing CSU. However, this may require further research and more data to verify. Our study also found that nucleotide metabolites such as adenine, adenosine, and inosine were up-regulated in CSU patients. Adenine, adenosine, and inosine are all key metabolites in purine metabolism. A study found that the level of inosine increased while the level of uric acid, the end product of purine metabolism, decreased in a mouse model of allergic asthma (Yu et al., 2016), which is similar to our study, but we did not find changes in uric acid. Another study showed that inflammatory cells in a mouse model of allergic asthma can induce the breakdown of ATP, resulting in increased levels of adenine and adenosine (Moon et al., 2010). Therefore, the above metabolite changes may be related to the inflammatory reaction, which in turn confirms that the inflammation is involved in the pathogenesis of CSU.

Due to the limited sample size and a single-center study, there are potential errors in the sample collection process, and confounding factors such as age, gender, BMI, ethnicity, region, and dietary habits cannot be controlled for matching. For example, CSU patients typically follow a low-protein diet, and changes in long-term eating habits may result in changes in microbiota. Also, metabolomics is impacted by factors such as age, sleep, circadian clock, and exercise, and almost half of the patients in our research had poor sleep quality, so these aspects must be considered. Therefore, this study has great limitations, and more research is needed to verify it. The above factors may also be the reason why our results differ from other studies. At the same time, due to the limitations of research methods, we cannot obtain information on fungi and viruses in the gut.

In conclusion, this study combined 16S gene sequencing and serum metabolomics to identify differences in gut microbiota and serum metabolites between CSU patients and normal controls, revealing that these differences may play a role in immune dysregulation and inflammation in the pathogenesis of CSU patients, providing more data for the study of CSU and gut microbiota. But more research is still needed to further define the exact microbiota that play a key role in CSU and its impact on the host.

## Data availability statement

The datasets presented in this study can be found in online repositories. The names of the repository/repositories and accession number(s) can be found below: NCBI, The accession number: PRJNA901136.

## Ethics statement

The studies involving human participants were reviewed and approved by Ethics Review Committee of the First Affiliated Hospital of Anhui Medical University. The patients/participants provided their written informed consent to participate in this study.

## Author contributions

Design of the study: ZL, and ZXW. Methodology: ZL, TW and ZXW. Collected samples and made clinical records: ZL, PLW, TW and XRT. Data curation: ZL, CHZ and ZXJ. Writing—original draft preparation: ZL and ZXJ. Writing—review and editing: ZL, XRT, and ZXW. All authors contributed to the article and approved the submitted version.
